# Neuronal DJ-1 regulates microglial activation in Parkinson’s disease

**DOI:** 10.4103/NRR.NRR-D-24-01047

**Published:** 2025-03-25

**Authors:** Aonan Zhao, Yanfei Ding, Min Zhong, Mengyue Niu, Lingbing Wang, Yang Jiao, Jun Liu, Yuanyuan Li

**Affiliations:** 1 Department of Neurology and Institute of Neurology, Ruijin Hospital Affiliated to the Shanghai Jiao Tong University School of Medicine, Shanghai, China; 2 Co-innovation Center of Neuroregeneration, Nantong University, Nantong, Jiangsu Province, China

**Keywords:** ADAM10/17 activity, central nervous system, endoplasmic reticulum, immune intervention, microglia, neuroinflammation, neuronal chemokines, neuronal DJ-1, *PARK7*, Parkinson’s disease

## Abstract

DJ-1, also known as Parkinson’s disease protein 7 (*PARK7*), is a multifunctional protein that plays an important role in oxidative stress regulation and neuroprotection. Previous studies have shown that DJ-1 affects early-onset Parkinson’s disease by regulating neuroinflammation, but its specific mechanism remains unclear. The study investigated the role of DJ-1 in mediating microglia–neuron communication to identify potential therapeutic targets for neuroinflammation in Parkinson’s disease. In this study, we observed a significant decrease in the levels of C-X3-C motif chemokine ligand 1 (CX3CL1) in *Park7* knockout mice and SH-SY5Y cells with *Park7* knockdown. Protein microarray analysis and validation using GEO datasets confirmed that knockout of the *Park7* gene led to downregulation of CX3CL1 and two other chemokines, namely monocyte chemoattractant protein-1 and interleukin-8. Further investigation revealed that *Park7* deficiency reduced the processing of a disintegrin and metalloproteinase domain-containing protein 10 (ADAM10) in the neuronal endoplasmic reticulum of both mice and SH-SY5Y cells, thereby decreasing CX3CL1 secretion. This subsequently led to abnormal microglial activation, with a shift toward the proinflammatory M1 phenotype, exacerbating neuroinflammatory responses. These effects were mitigated by exogenous CX3CL1 administration. Concurrently, exogenous CX3CL1 improved motor function in Parkinson’s disease model mice with the *Park7* knockout, promoting survival of tyrosine hydroxylase-positive neurons in the substantia nigra and reducing Iba-1-positive microglial activation. These findings demonstrate that DJ-1 exerts neuroprotective effects on dopaminergic neurons by suppressing microglial activation through CX3CL1 regulation, suggesting that targeting the DJ-1/CX3CL1 axis may represent a novel therapeutic strategy for modulating neuroinflammation and protecting dopaminergic neurons.

## Graphical abstract

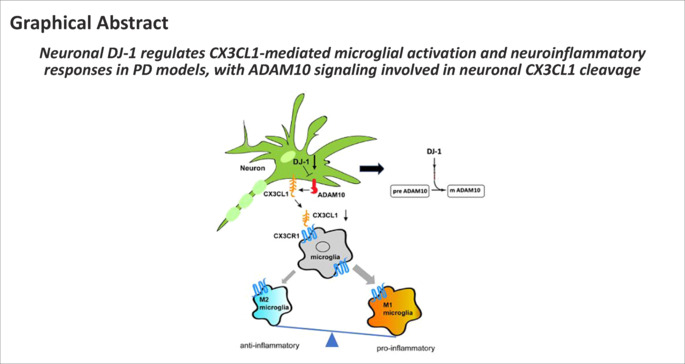

## Introduction

Parkinson’s disease (PD) is a neurodegenerative condition that affects approximately 1% of people aged 60 and above (Savica et al., 2013). The disease is primarily defined by the gradual loss of dopaminergic neurons in the substantia nigra (SN) and the presence of Lewy bodies, which are intracellular protein aggregates predominantly comprising α-synuclein (Barzilai and Melamed, 2003). These pathological hallmarks contribute significantly to the motor and cognitive impairments observed in PD patients. Microglial inflammation within the SN is increasingly recognized as a pivotal pathological feature associated with neurodegeneration in PD (Hu et al., 2015; Le et al., 2016). Mounting evidence suggests that dysfunctional interactions between microglia and neurons may exacerbate disease progression, although the precise mechanisms remain poorly understood.

DJ-1, also known as Parkinson’s disease protein 7, is encoded by PARK7 and is associated with a rare form of early-onset PD inherited through an autosomal recessive pattern (Bonifati et al., 2003). This multifunctional protein is crucial for preserving mitochondrial stability and managing cellular responses to oxidative stress (Taira et al., 2004; Abramov et al., 2017). In recent years, attention has increasingly shifted toward the role of DJ-1 in modulating neuroinflammation, suggesting it may significantly influence the underlying pathological mechanisms of PD (Yang et al., 2020a). Studies using DJ-1 (*PARK7*) knockout (KO) models have highlighted that the loss of DJ-1 significantly enhances microglial activation via mechanisms involving the NOD-like receptor family pyrin domain containing 3 (NLRP3) inflammasome and signal transducer and activator of transcription 1 (STAT1) signaling pathways (Kim et al., 2013; Choi et al., 2019). By influencing the release of inflammatory mediators, DJ-1 plays an essential role in promoting the anti-inflammatory functions of microglia (Kim et al., 2014; Ji et al., 2020). Despite these findings, the detailed pathways through which neuronal DJ-1 modulates microglia-driven inflammatory responses in PD remain poorly understood, necessitating further research to pinpoint therapeutic opportunities.

Chemokines play a pivotal role in mediating the communication between neurons and microglia during the development of PD, facilitating processes such as microglial phagocytosis and activation (Liu et al., 2019a). These molecules serve as crucial mediators in the inflammatory responses within the central nervous system (CNS), influencing disease progression. Preliminary studies have suggested a potential link between DJ-1 and chemokine regulation. Specifically, studies have observed a significant reduction in the mRNA levels of C-X3-C motif chemokine ligand 1 (*CX3CL1*) in *Park7* KO mice, coupled with aberrant macrophage activity in liver progenitor cells (Chen et al., 2016). Despite these findings, few reports have comprehensively detailed the regulatory influence of neuronal DJ-1 on chemokines in the context of PD.

CX3CL1, the sole member of the CX3C chemokine family, is a transmembrane protein whose expression is localized to specific neurons within the CNS (Jones et al., 2010). Upon proteolytic cleavage from the cell membrane, CX3CL1 fragments bind to microglial CX3CR1 receptors, thereby modulating neuroinflammatory responses (Garton et al., 2001; Hickman et al., 2018). This interaction plays a crucial role in regulating microglial activation and subsequent immune responses in neurodegenerative diseases such as PD. Emerging research has highlighted the promise of CX3CL1 for mitigating neuroinflammation through modulation of microglial activity in PD (Subbarayan et al., 2022). The observed decrease in CX3CL1 levels in *Park7*-deficient models prompts further investigation into the specific mechanism by which *Park7* impacts CX3CL1 expression and its implications for PD pathogenesis. Within the CNS, a disintegrin and metalloproteinase domain-containing protein (ADAM)10 and ADAM17, members of the ADAM family of metalloproteases, play crucial roles in the release of CX3CL1 from cell membranes in various pathological contexts (Hundhausen et al., 2003; Jones et al., 2013; O’Sullivan et al., 2016; Hattori et al., 2017). Research indicates that the endoplasmic reticulum (ER) is critical for the maturation and activity of a disintegrin and metalloproteinase domain-containing protein 10/17 (ADAM10/17) (Hundhausen et al., 2003). Loss of DJ-1 function has been associated with compromised ER function, potentially affecting ADAM-mediated processing of CX3CL1 (Hattori et al., 2017; Urano et al., 2018). Therefore, exploring the correlation between neuronal DJ-1 and ADAM10/17 could provide insights into how DJ-1 influences CX3CL1 shedding and subsequent microglial activation in PD.

Building on these findings, we hypothesized that diminished DJ-1 expression in neurons hinders the proper maturation and membrane shedding of CX3CL1, likely through adverse effects on ADAM10 functionality within the ER. This disruption could lead to aberrant microglial activation and phenotypic changes in a proinflammatory CNS environment, potentially exacerbating neurodegenerative processes in PD. Here, we investigated the role of DJ-1 in mediating microglia–neuron communication to identify potential therapeutic targets for neuroinflammation in PD. By elucidating the complex interactions involving DJ-1, ADAM10/17, and CX3CL1, we aim to advance strategies for modulating microglial responses, reducing neuroinflammation, and significantly enhancing neuroprotection in PD.

## Methods

### Protein-chip and GEO database analysis

The expression profiles of 15 chemokine-related genes were analyzed using a mouse custom polymerase chain reaction (PCR) array kit (Biotnt, Hangzhou, Zhejiang, China), while chemokine levels were assessed with a protein-chip array (Raybiotech, Norcross, GA, USA), each in accordance with the respective manufacturer’s instructions. The protein array results were scanned using an ImageQuant LAS4000 Scanner (Cytiva, Marlborough, MA, USA). For additional mRNA expression analysis, transcriptomic data were retrieved from the GEO database (GSE17204; https://www.ncbi.nlm.nih.gov/geo/query/acc.cgi?acc=GSE17204; Foti et al., 2010). The GEO2R online analysis tool (https://www.ncbi.nlm.nih.gov/geo/geo2r/) was used to compare mRNA expression levels between *Park7*-silenced and control neuron samples in SH-SY5Y cells. To further confirm, we used the GEO database (GSE114918; https://www.ncbi.nlm.nih.gov/geo/query/acc.cgi?acc=GSE114918; Peng et al., 2024) for RNA sequencing data on dopamine neurons from the SN pars compacta (SNc) and ventral tegmental area (VTA) that were selectively extracted from postmortem brain samples of PD patients and healthy controls (HCs) using laser capture microdissection techniques. The GEO2R online analysis tool was used to compare the HCs and PD patients. A heatmap illustrating the differentially expressed genes was generated using the GEO2R online tool and the R language package (https://cran.r-project.org/).

### Human blood samples

Blood samples were obtained from a cohort of PD patients (age: 60–90 years) of both sexes and age-matched controls who visited the Neurology Clinic at Ruijin Hospital between 2018 and 2020. All PD patients were clinically diagnosed with sporadic PD of comparable severity and had no comorbid neurological disorders. Control participants were confirmed to have no history of neurological conditions. Collected blood samples were centrifuged, and the plasma-containing supernatants were aliquoted into separate vials and stored at –80°C for batch analysis. The PD staging assessment was based on the Hoehn and Yahr (H-Y) scale (Li et al., 2022). Patients classified as being in the early stages of PD scored ≤ 2.5 on the H-Y scale and had been living with the disease for < 5 years. All procedures were performed in strict accordance with the *Declaration of Helsinki*, and the study was approved by the Ruijin Hospital Ethics Committee (2016YFC1306505; June 13, 2018). Informed consent was obtained from all participants.

### Animals

Fifty male *Park7* KO (*Park7*^–/–^) mice (B6.cg-Park7tm1shn/J, RRID: IMSR_JAX:006577) and 50 male wild-type (WT) mice (aged 3–6 months, weighting 32–37 g) with an identical genetic background, which were chosen to avoid estrogen-related variability, were obtained from the Jackson Laboratory (Bar Harbor, ME, USA). The mice were kept in a strictly controlled, pathogen-free environment at the Experimental Animal Center of Shanghai Jiao Tong University School of Medicine to ensure optimal health and experimental consistency. Neonatal C57BL/6 mice (age: **~**7 days) were provided by Shanghai Model Organisms Center (Shanghai, China; animal license No. SYXK (Hu) 2018-002). The animal experiments were carried out following the ethical guidelines established by the Laboratory Animal Ethics Committee of Shanghai Jiao Tong University School of Medicine on February 25, 2019.

### Cell isolation, culture, and transfection

This study employed the following cell types: BV2 immortalized murine microglial cells (Cell Bank of Chinese Academy of Sciences; identifier: CSTR:19375.09.3101MOUSCSP5208, Cat# SCSP-5208, RRID: CVCL_0182), human SH-SY5Y neuroblastoma cells (Cell Bank of Chinese Academy of Sciences; identifier: CSTR:19375.09.3101HUMSCSP5014, Cat# SCSP-5014, RRID: CVCL_0019), primary microglia, and primary neurons.

For gene knockdown, cells in the logarithmic growth phase were transfected with small interfering RNAs (siRNAs) against *Park7* or ADAM10 (Guangzhou Ruibo Biotech Co., Ltd., Guangzhou, China) using Lipofectamine 2000 transfection reagent (Invitrogen, Thermo Fisher Scientific, Waltham, MA, USA).

Co-cultures of SH-SY5Y and BV2 cells were carried out using a transwell system (Corning, Corning, NY, USA) with a 0.4-µm pore polyester membrane to separate the two cell types. Briefly, BV2 cells were seeded in the upper chamber, and SH-SY5Y cells were placed in the lower chamber of a 6-well plate. The co-culture was maintained for 24 hours to allow for interaction and release of cytokines.

For the rescue experiments, cells were co-transfected with siRNA and a plasmid directing overexpression of either *Park7* or ADAM10. After a 48-hour incubation, cells were harvested for subsequent analyses.

Primary microglia and primary neurons were isolated from the brains of neonatal C57BL/6 mice anesthetized with a 50-mg/kg intraperitoneal (i.p.) injection of pentobarbital (Sigma Aldrich, St. Louis, MO, USA) using magnetic-activated cell sorting technology (Miltenyi Biotec, Bergisch Gladbach, Germany), in accordance with the manufacturer’s protocol. Briefly, single-cell suspensions extracted from the brains of 12 neonatal mice were incubated with magnetic beads coated with an anti-CD11b antibody (BioLegend, San Diego, CA, USA). The labeled cells were then passed through a magnetic-activated cell sorting column to selectively isolate CD11b^+^ microglia, which were seeded in 24-well plates and maintained in Dulbecco’s modified Eagle’s medium/nutrient mixture F-12 complete medium (Life Technologies, Carlsbad, CA, USA). After 2–4 days of culture, the microglia were harvested for experimental use. The primary neurons were plated on poly-D-lysine-coated culture dishes and cultured in Neurobasal medium supplemented with B27 and GlutaMAX (Thermo Fisher Scientific, Waltham, MA, USA) at 37°C in a 5% CO_2_ incubator. The medium was changed regularly, and the cells matured within 7–10 days.

### Surgical procedure and behavioral analysis

For PD modeling, mice were given i.p. injections of 1-methyl-4-phenyl-1,2,3,6-tetrahydropyridine (MPTP; Sigma Aldrich) at a dose of 30 mg/kg or Saline every other day over a 30-day period. During the modeling process, mice were randomly bilateral stereotactic injection of 400 pmol (200 nL) CX3CL1 (Peprotech, Rocky Hill, CT, USA) into the SNc (anteroposterior, –2.8 mm; mediolateral, ±1.4 mm; dorsoventral, –4.6 mm (Carty et al., 2010) or 200 nL of sterile saline and divided into four groups: Control group, Saline + CX3CL1 group (CX3CL1 stereotactic injection), MPTP + CX3CL1 group (i.p., MPTP + CX3CL1 stereotactic injection), and MPTP + Saline group (i.p., MPTP).

Three days after the final MPTP injection, the mice underwent behavioral tests to assess overall balance and motor coordination. The rotarod assay was conducted using an accelerating rotarod apparatus (Ugo Basile, Gemonio, Varese, Italy) (Rogers et al., 2011), and the latency to fall from the accelerating rod was recorded to assess motor coordination and balance. The open field test was conducted in a square field measuring 50 cm × 50 cm × 30 cm, following the method outlined by Malloul et al. (2017), and locomotor activity and exploratory behavior were measured by total distance traveled. All tissues were harvested after completing the treatment and behavioral assessments, and subsequently analyzed.

### DJ1 overexpression via brain-targeted viral vector injection

The plasmid pAAV2-CBA-HA-DJ-1-WPRE expresses HA-tagged human DJ-1 was cloned from SH-SY5Y cell RNA via reverse transcription. rAAV2 particles were generated by co-transfecting the AAV2 vector plasmid into 293T cells and harvested after 48–72 hours, followed by purification via iodixanol gradient ultracentrifugation. Viral titers (vg/mL) were measured by optical density. Bilateral substantia nigra injections (2 μL per side; 0.2 μL/min) were stereotaxically targeted (coordinates from Bregma: 3.0 mm anterior-posterior, ±1.2 mm medial-lateral, 4.2 mm dorsal-ventral).

### Tissue preparation

For immunofluorescence staining analysis, 12- to 16-week-old WT and *Park7*^–/–^ littermates were euthanized with a 50 mg/kg i.p. injection of pentobarbital. The SN brains were removed and immersed in 4% paraformaldehyde for 12 hours, then transferred to a 30% sucrose solution at 4°C for a minimum of 16 hours to achieve proper cryoprotection. Coronal brain sections (thickness: 25 μm) were cut and preserved in cryoprotectant at 4°C.

To prepare tissue lysates for western blotting and enzyme-linked immunosorbent assays (ELISAs), brain tissues, including the SN, were homogenized using an electric tissue homogenizer in ice-cold radioimmunoprecipitation assay buffer at a 1:10 weight-to-volume ratio. The buffer was supplemented with protease and phosphatase inhibitors (Bimake, Houston, TX, USA) to preserve protein integrity. Protein concentrations were measured using a bicinchoninic acid assay kit (Pierce, Thermo Fisher Scientific).

Subcellular fractionation of cells and brain tissues was carried out following a published protocol (Liu et al., 2019b). Briefly, cells and brain tissues were gently disrupted by repeated strokes using a Dounce homogenizer (Wheaton, Millville, NJ, USA). The supernatant was then centrifuged at 20,000 × *g* for 30 minutes at 4°C to isolate plasma membrane fractions. The supernatant was further centrifuged (SW50.1 rotor; Beckman Coulter, Brea, CA, USA) at 4°C to separate the ER (pellet) from the cytosolic fraction (supernatant).

For *in vitro* staining, SH-SY5Y cells were fixed by immersion in 4% paraformaldehyde for 15 minutes. For biochemical experiments, BV2 cells, primary microglia, and SH-SY5Y cells were incubated on ice in radioimmunoprecipitation assay lysis buffer with phenylmethylsulfonyl fluoride (Beyotime, Shanghai, China) to ensure efficient protein extraction.

### Immunofluorescence staining

For dopaminergic neuron labeling, sections were treated with chicken anti-tyrosine hydroxylase (TH) polyclonal antibody (1:4000 dilution; Abcam, Cambridge, Cambridgeshire, UK; Cat# ab76442, RRID: AB_297840) and ADAM10 antibody (1:100; Proteintech, Wuhan, Hubei, China; Cat# 25900-1-AP, RRID: AB_288029) at 4°C for 24 hours, followed by incubation with Alexa Fluor 594-conjugated goat anti-chicken IgG (1:1000; Abcam; Cat# ab150172, RRID: AB_3662878) at 4°C overnight. To visualize activated microglia, sections were treated with rabbit anti-ionized calcium binding adapter molecule 1 (Iba-1; 1:200; Wako, Osaka, Japan; Cat# 019-19741, RRID: AB_839504) at 4°C for 24 hours, followed by Alexa Fluor 488-conjugated donkey anti-rabbit IgG (1:1000; Abcam; Cat# ab150077, RRID: AB_2630356) at 4°C overnight. After incubation with secondary antibodies for 24–48 hours, the sections were mounted using an antifade medium containing 4’,6-diamidino-2-phenylindole (Beyotime) to visualize cell nuclei. Cell density in the SN was measured using a Zeiss Mirax slide scanner (Oberkochen, Baden-Württemberg, Germany), and the data were analyzed with Zeiss NeuroQuant IAE software (Oberkochen). The stereological counting method was used to accurately assess the number of TH-positive neurons (Vivacqua et al., 2020). To ensure representativeness and minimize potential double-counting, we selected one of every three consecutive sections for staining and analysis, covering the entire SN.

### Western blot analysis

Proteins were separated by sodium dodecyl sulfate-polyacrylamide gel electrophoresis on gels from Bio-Rad (Hercules, CA, USA) and then transferred to a polyvinylidene fluoride membrane for immunodetection. The membrane was blocked with 5% bovine serum albumin in non-fat dry milk (Bio-Rad) to prevent non-specific binding. Primary antibodies against the following were incubated with the membrane overnight at 4°C: DJ-1 (rabbit IgG, 1:2000; CST, Danvers, MA, USA; Cat# 5933, RRID: AB_11179085), CX3CL1 (mouse IgG, 1:500; R&D Systems, Minneapolis, MN, USA; Cat# MAB3652, RRID: AB_2087144), glyceraldehyde 3-phosphate dehydrogenase (rabbit IgG, 1:3000; CST; Cat# 2118, RRID: AB_561053), Na-K ATPase (rabbit IgG, 1:1000; CST; Cat# 3010, RRID: AB_2060983), ADAM10 (rabbit IgG, 1:500; R&D Systems; Cat# MAB1427, RRID: AB_2223057), and ADAM17 (rabbit IgG, 1:500; R&D Systems; Cat# AF9301, RRID: AB_10891879). Following this incubation, the membrane was washed and incubated with horseradish peroxidase-conjugated secondary antibodies to goat anti-rabbit IgG (1:10,000; Jackson ImmunoResearch, West Grove, PA, USA; Cat# 111-035-144, RRID: AB_2307391) or goat anti-mouse IgG (1:10,000; Jackson ImmunoResearch; Cat# 111-035-003, RRID: AB_2313567). Protein bands were visualized using a chemiluminescence method, and images were captured using the Tanon 5200 Multi Chemiluminescent Imaging System (Shanghai, China). Signal intensities (optical densities) were measured and quantified with ImageJ software (v1.8.0; National Institutes of Health, Bethesda, MD, USA; Schneider et al., 2012). Protein levels were quantified by densitometric analysis and normalized to the stable housekeeping protein glyceraldehyde 3-phosphate dehydrogenase (GAPDH) to correct for loading variations.

### Enzyme-linked immunosorbent assay

The levels of CX3CL1, interleukin (IL)-6, and IL-1β were analyzed using ELISA kits following standardized procedures. For each measurement, tissue lysates (100 µg) or cell culture supernatants (500 µL) were loaded into ELISA plate wells. After incubation at 4°C overnight, the optical density (OD) was measured using a BioTek plate reader (Winooski, VT, USA), and sample concentrations were calculated using the standard curve.

### Real-time polymerase chain reaction

Mouse brain tissue was homogenized using TRIzol reagent (Thermo Fisher Scientific), and total RNA was isolated from the homogenate in accordance with the manufacturer’s guidelines. Total RNA was quantified, and 1 μg was reverse transcribed into complementary DNA (cDNA) using a TaKaRa kit (Takara, Kusatsu, Shiga, Japan). The cDNA was combined with real-time PCR premix (Biotnt) and analyzed using a PCR array. The relative expression levels of the indicated mRNAs were quantified using real-time PCR performed on an Applied Biosystems 7500 fluorescence quantitative PCR (qPCR) system (Thermo Fisher Scientific) in a 20-μL reaction containing SYBR Green master mix (Takara) and the following primers: *Il-1*β, forward 5′-CTG TGA CTC ATG GGA TGA TGA TG-3′, reverse 5′-CGG AGC CTG TAG TGC AGT TG-3′; *Il-6*, forward 5′-CTG CAA GAG ACT TCC ATC CAG-3′, reverse 5′-AGT GGT ATA GAC AGG TCT GTT GG-3′; and *Tnf-α*, forward 5′-CTG AAC TTC GGG GTG ATC GG-3′, reverse 5′-GGC TTG TCA CTC GAA TTT TGA GA-3′. *GAPDH* served as the reference gene for normalization.

### Flow cytometry analysis

Flow cytometry was employed to evaluate the expression of CD11b in microglia and CD86/CD206 in BV2 cells. Single-cell suspensions prepared from the SN of three mice each in the Park7 KO and WT groups were passed through 40-μm cell strainers to remove debris and clumped cells, followed by three washing steps to ensure high sample purity. To determine the efficiency of isolating primary microglia, single-cell suspensions were collected both before and after magnetic-activated cell sorting, as per established protocols. To confirm the successful enrichment and purity of the microglial populations, the cells were labeled with FITC-conjugated anti-CD11b antibodies (BioLegend) and analyzed via flow cytometry. BV2 cells used for flow cytometry were collected in ice-cold phosphate-buffered saline and maintained at 4°C to preserve cell integrity. BV2 cells were stained with phycoerythrin (PE)-labeled anti-CD86 and allophycocyanin (APC)-labeled anti-CD206 antibodies (BioLegend) to detect M1 phenotype markers and M2 phenotype markers, respectively. Flow cytometry data were analyzed using CytExpert software (Beckman Coulter) to assess the expression of these polarization markers, characterizing the inflammatory status of microglial cells.

### Statistical analysis

GraphPad Prism 7.0.1 software (GraphPad Software, Boston, MA, USA, www.GraphPad.com) was used to perform statistical analyses of group differences. Student’s *t*-tests were used for two-group comparisons, one-way analysis of variance followed by Tukey’s *post hoc* test was applied for comparisons involving more than two groups, and Pearson correlation was applied for correlation analysis. Data are expressed as means ± standard error of the mean (SEM) from three independent experiments. A *P* value of < 0.05 was considered to indicate statistical significance.

## Results

### DJ-1 is correlated with the release of CX3CL1

**Figure 1:**
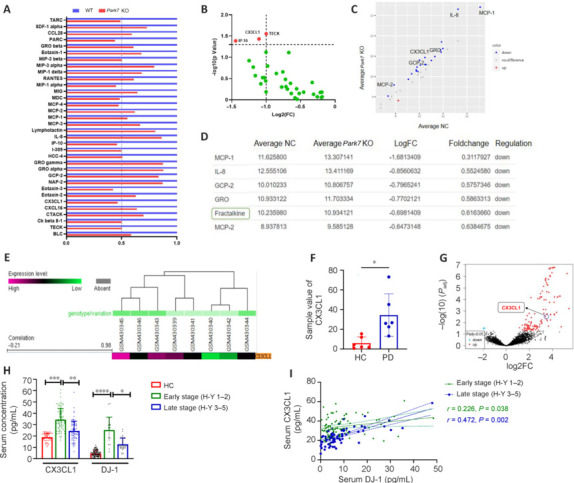
DJ-1 is positively correlated with CX3CL1 release. (A, B) PCR analysis of the expression of 34 chemokines in primary mesencephalic neurons from *Park7* KO and WT mice, showing reductions in IP-10, CX3CL1, and TECK in *Park7* KO mice. (C, D) Protein microarray analysis of neuron supernatants from NC and *Park7* KO mice, showing suppressed levels of MCP-1, IL-8, GCP-2, CX3CL1, and MCP-2 in Park7 KO mice. (E) CX3CL1 levels in SH-SY5Y cells, showing downregulation under *Park7* depletion (GEO: GSE17204). (F, G) RNA sequencing of PD patient and HC brain tissues (GEO: GSE114918), showing high CX3CL1 expression in the SNc. (H) Serum levels of DJ-1 and CX3CL1, showing a rise in early PD and a decline in advanced PD: for CX3CL1, *n* = 60 in the HC, *n* = 84 in the early stage (H-Y 1–2), and *n* = 76 in the late stage (H-Y 3–5) groups; for DJ-1, *n* = 98 in the HC, *n* = 23 in the early stage (H-Y 1–2), and *n* = 29 in the late stage (H-Y 3–5) groups. (I) Positive correlation between serum levels of DJ-1 and CX3CL1 across PD stages: *n* = 160 in the DJ-1, *n* = 84 in the early stage (H-Y 1–2), and *n* = 76 in the late stage (H-Y 3–5) groups. Data expressed as means ± SEM; *P < 0.05, **P < 0.01, ***P < 0.001, ****P < 0.0001, analyzed using one-way analysis of variance followed by Tukey’s *post hoc* test. CX3CL1: Chemokine (C–X3–C motif) ligand 1; DJ-1: protein deglycase DJ-1; GCP-2: granulocyte chemotactic protein-2; HC: healthy control; H-Y: Hoehn and Yahr; IL-8: interleukin 8; IP-10: interferon gamma-induced protein 10; KO: knockout; MCP-1: monocyte chemoattractant protein-1; MCP-2: monocyte chemoattractant protein-2; PCR: polymerase chain reaction; *Park7*: Parkinson’s disease protein 7; PD: Parkinson’s disease; SNc: substantia nigra pars compacta; TECK: thymus-expressed chemokine; VTA: ventral tegmental area; WT: wild type.

To investigate the role of DJ-1 in chemokine regulation, we analyzed a panel of 34 chemokines in primary mesencephalic neurons derived from *Park7* KO and WT mice. As illustrated in **Figure 1A** and **B**, the loss of *Park7* significantly diminished the expression levels of key chemokines, namely interferon gamma-induced protein 10 (IP-10), CX3CL1 (also known as fractalkine), and thymus-expressed chemokine (TECK) in *Park7* KO mice. Furthermore, a protein microarray chip analysis of the supernatant from primary mesencephalic neurons revealed that *Park7* KO resulted in the suppression of multiple chemokines, namely monocyte chemoattractant protein (MCP)-1, IL-8, granulocyte chemotactic protein 2 (GCP-2), CX3CL1, and MCP-2 (**Figure 1C** and **D**). Among these, CX3CL1 emerged as a particularly noteworthy candidate and was thus further validated through expression profiling in *Park7*-depleted SH-SY5Y neuroblastoma cells, using data from the GEO database (GSE17204; **Figure 1E**).

To explore the clinical relevance of these findings, we also analyzed CX3CL1 expression in PD patients and HC (Healthy Control) brain tissues using the GEO database (GSE114918). RNA sequencing of dopamine neurons in postmortem brain tissues confirmed that CX3CL1 was highly expressed in the SNc samples from individuals with PD (**Figure 1F** and **G**). Next, peripheral blood samples from PD patients and HCs were analyzed to determine the correlation between DJ-1 and CX3CL1 in PD pathology. The results demonstrated that, compared with the levels in HC, serum levels of DJ-1 and CX3CL1 in PD patients initially increased during the early stages of PD (*P* < 0.001), subsequently decreasing as the disease progressed (*P* < 0.01; **Figure 1H**). Furthermore, positive correlations between serum levels of DJ-1 and CX3CL1 were observed at different stages of the disease (*P* < 0.01; **Figure 1I**), further supporting the involvement of DJ-1 in modulating CX3CL1 expression and potentially in PD pathology.

### DJ-1 mediates CX3CL release *in vivo* and *in vitro*

**Figure 2:**
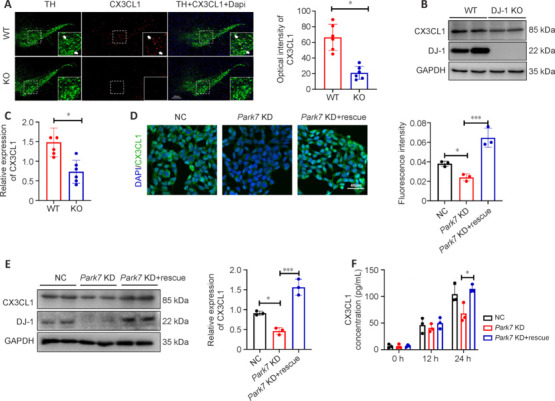
DJ-1 regulates CX3CL1 expression and release in neuronal cells. (A) Immunofluorescence analysis of CX3CL1 (red, Alexa Fluor 594) intensity in TH-positive (green, Alexa Fluor 488) neurons of *Park7* KO mice and WT mice. Scale bar: 200 μm. (B, C) Western blotting of CX3CL1 protein in the SN of *Park7* KO and WT mice. (D) Immunofluorescence of CX3CL1 (green, Alexa Fluor 488) in SH-SY5Y cells, with and without *Park7* KD and rescue. Scale bar: 40 μm. (E) Western blotting and quantitative analysis of CX3CL1 protein expression in SH-SY5Y cells, with and without *Park7* KD and rescue. (F) Enzyme-linked immunosorbent assay of CX3CL1 secretion in SH-SY5Y cells, with and without *Park7* KD and rescue. Data expressed as means ± SEM (*n* = 6 per group *in vivo*; *n* = 3 per group *in vitro*); *P < 0.05, ***P < 0.001, analyzed using Student’s *t*-tests (A, C) or one-way analysis of variance followed by Tukey’s *post hoc* test (D–F). CX3CL1: Chemokine (C–X3–C motif) ligand 1; DJ-1: protein deglycase DJ-1; GAPDH: glyceraldehyde 3-phosphate dehydrogenase; KD: knockdown; KO: knock out; NC: negative control; *Park7*: Parkinson’s disease protein 7; SN: substantia nigra; TH: tyrosine hydroxylase; WT: wild type.

To ascertain whether DJ-1 influences the release of CX3CL1 *in vivo*, we generated *Park7* KO mice and examined changes in CX3CL1 expression. *Park7* KO mice exhibited lower fluorescence intensities of CX3CL1 in TH-positive neurons compared with WT mice (**Figure 2A**). *Park7* KO mice also showed a significant reduction in the level of CX3CL1 protein in the SN compared with WT mice, as illustrated in **Figure 2B** and **C**. To validate the regulatory role of neuronal DJ-1 on CX3CL1 secretion, we established SH-SY5Y neuroblastoma cells with siRNA-mediated knockdown of *Park7* (*Park7* KD) and a *Park7* KD + rescue model, in which SH-SY5Y cells were co-transfected with *Park7* KD siRNA and a *Park7* overexpression plasmid. Immunofluorescence analysis showed that the reduction in CX3CL1 intensity observed in Park7 KD cells was significantly reversed in *Park7* KD + rescue cells, demonstrating the role of DJ-1 in maintaining CX3CL1 expression (**Figure 2D**). Consistently, western blotting showed a notable reduction in CX3CL1 protein levels in *Park7* KD cells that was partially restored in *Park7* KD + rescue cells (**Figure 2E**). Furthermore, ELISAs of culture supernatants showed a partial restoration of CX3CL1 secretion in *Park7* KD + rescue cells compared with the findings in *Park7* KD cells, further supporting the involvement of *Park7* in CX3CL1 release (**Figure 2F**). This series of experiments underscored the critical role of *Park7* in modulating CX3CL1 expression and release in neuronal cells.

### DJ-1 regulates CX3CL1 secretion through ADAM10, not ADAM17

**Figure 3:**
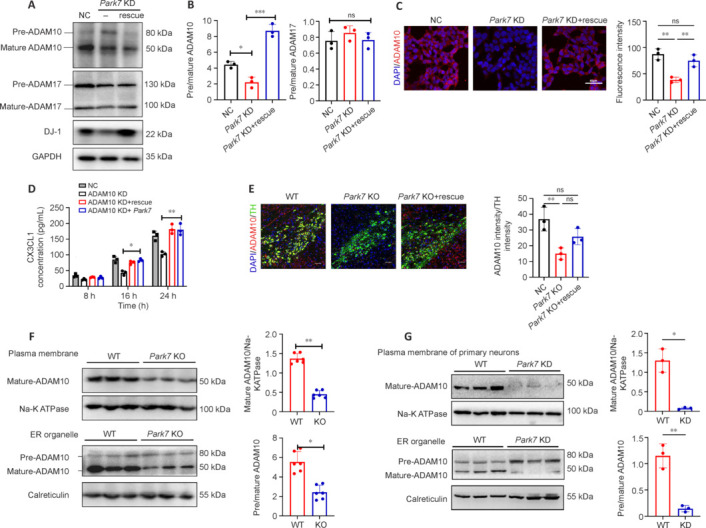
DJ-1 regulates ADAM10 maturation and trafficking to facilitate CX3CL1 release. (A, B) Western blot analysis showing the ratios of mature to precursor ADAM10 and ADAM17 in SH-SY5Y cells, with and without *Park7* KD and ADAM10 rescue. (C) Immunofluorescence of ADAM10 (red, Alexa Fluor 594) in SH-SY5Y cells, with and without *Park7* KD and ADAM10 rescue. Scale bar: 40 μm. (D) Enzyme-linked immunosorbent assay of CX3CL1 secretion in SH-SY5Y cells, with ADAM10 KD and with ADAM10 rescue or with *Park7* in neurons. (E) Immunofluorescence of TH-positive neurons (green, Alexa Fluor 488) and ADAM10 (red, Alexa Fluor 594) in the SN of PD model brains from WT, *Park7* KO, and *Park7* KO + rescue mice. ADAM10 expressed in neurons: ADAM10 merged TH created yellow. Scale bars: 50 μm. (F, G) Western blotting of ER and plasma membrane fractions of brain tissues of WT and *Park7* KO mice (F) and primary neurons, with and without *Park7* KD (G), showing mature ADAM10 levels at the plasma membrane. Data expressed as means ± SEM (*n* = 6 per group *in vivo* experiments, *n* = 3 per group *in vitro*); *P < 0.05, **P < 0.01, ***P < 0.001, analyzed using one-way analysis of variance followed by Tukey’s *post hoc* test (B–E) or Student’s *t*-tests (F, G). ADAM10: A metalloproteinase domain-containing protein 10; ADAM17: metalloproteinase domain-containing protein 17; DJ-1: protein deglycase DJ-1; ER: endoplasmic reticulum; GAPDH: glyceraldehyde 3-phosphate dehydrogenase; KD: knockdown; KO: knock out; NC: negative control; ns: no significance; *Park7*: Parkinson’s disease protein 7; PD: Parkinson’s disease; TH: tyrosine hydroxylase; WT: wild type.

Next, we sought to explore the mechanism by which DJ-1 regulates CX3CL1. The extracellular domain of CX3CL1 is cleaved by ADAM10 in the absence of exogenous stimulation or by ADAM17 upon exogenous stimulation, after which it is released into the extracellular environment (Lammich et al., 1999; Kärkkäinen et al., 2000). To verify the hypothesis that neuronal DJ-1 interferes with CX3CL1 release by affecting the shear-mediated maturation of ADAM10 or ADAM17, we performed western blotting analysis of ADAM10 and ADAM17 under *Park7* KD and *Park7* KD + rescue in SH-SY5Y cells. Compared with the findings in the *Park7* non-knockout group (NC group), the ratio of the mature to precursor forms of ADAM10 was significantly decreased, while ADAM17 levels remained unchanged, in the *Park7* KD group (*P* < 0.001; **Figure 3A** and **B**). Furthermore, immunofluorescence showed reduced ADAM10 intensity in *Park7* KD group cells, which was restored in the rescue group cells (*P* < 0.001; **Figure 3C**). This confirmed that DJ-1 plays a crucial role in regulating ADAM10. Next, to examine the role of ADAM10 in CX3CL1 shedding in neurons, we used ELISAs to assess CX3CL1 secretion in cells pretreated with ADAM10 siRNA (ADAM10 KD) followed by a 24-hour incubation. Subsequently, the cells were transfected with either ADAM10 (labeled as ‘rescue’) or *Park7* (labeled as ‘Park7’) plasmid for 6 hours. After transfection, we replaced the plasmid with cell culture medium and then measured the CX3CL1 levels at different time points. As expected, the group pretreated with ADAM10 siRNA showed significantly reduced CX3CL1 secretion compared with the NC group, whereas the rescue group showed restored CX3CL1 levels compared with the group at 12 and 24 hours (*P* < 0.05; **Figure 3D**). The maturation and cleavage of ADAM10 occur in the ER, where it is proteolytically activated during its forward transport along the secretory pathway and at the plasma membrane (Yang et al., 2020b). To further explore this in PD, we examined ADAM10 fluorescence intensity in TH-positive neurons of the SN in the mouse brain. Immunofluorescence analysis showed reduced ADAM10 expression in the *Park7* KO group compared with that in the WT group, which was restored in the *Park7* KO + rescue group (*P* < 0.001; **Figure 3E**). Next, we used density gradient centrifugation to isolate the ER and cell membranes of brain tissues from *Park7* KO and WT mice. The mature form of ADAM10 at the plasma membrane was reduced in Park7 KO mice compared with that in WT mice, potentially owing to impaired ER cleavage (**Figure 3F**). There were similar findings in primary neurons, where the *Park7* knockdown disrupted ADAM10 trafficking, and rescue restored its levels at the plasma membrane (**Figure 3G**). Taken together, these results demonstrated that *Park7* regulates ADAM10 maturation and trafficking, facilitating CX3CL1 release.

### DJ-1 inhibits microglia-induced inflammation via CX3CL1

**Figure 4:**
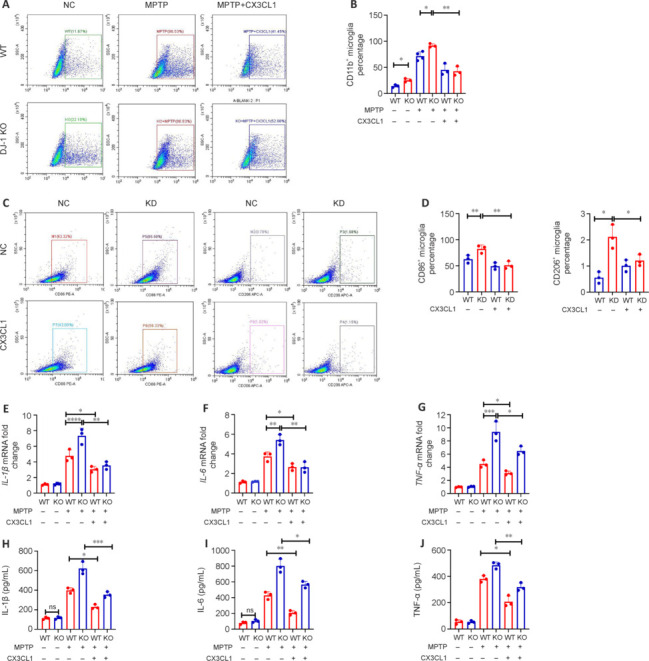
DJ-1 regulates microglial activation and proinflammatory responses through CX3CL1 release. (A, B) Flow cytometry analysis of CD11b-reactive microglia in the SN of WT and *Park7* KO mice, with and without MPTP induction and CX3CL1 treatment. (C, D) Flow cytometry analysis of the M1 marker CD86 and the M2 marker CD206 in BV2 microglia co-cultured SH-SY5Y neurons with and without *Park7* KD and CX3CL1 treatment. (E–G) qPCR analysis of the mRNA levels of *TNF-α*, *IL-6*, and *IL-1*β in SN tissue lysates of WT and *Park7* KO mice, with and without MPTP induction and CX3CL1 treatment. (H–J) Enzyme-linked immunosorbent assay of cytokines IL-1β, IL-6, and TNF-α in SN tissue lysates of WT and *Park7* KO mice, with and without MPTP induction and CX3CL1 treatment. Data expressed as means ± SEM (*n* = 3 per group); *P < 0.05, **P < 0.01, ***P < 0.001, analyzed by one-way analysis of variance followed by Tukey’s *post hoc* test. CD11b: Cluster of differentiation 11b; CD206: cluster of differentiation 206; CD86: cluster of differentiation 86; CX3CL1: chemokine (C–X3–C motif) ligand 1; DJ-1: protein deglycase DJ-1; GAPDH: glyceraldehyde 3-phosphate dehydrogenase; Iba-1: ionized calcium binding adapter molecule; IL-1β: interleukin-1 beta; IL-6: interleukin-6; KD: knockdown; KO: knock out; MPTP: 1-methyl-4-phenyl-1,2,3,6-tetrahydropyridine; NC: negative control; *Park7*: Parkinson’s disease protein 7; SN: substantia nigra; TH: tyrosine hydroxylase; TNF-α: tumor necrosis factor-α; WT: wild type.

To certify whether neuronal DJ-1 directly regulates microglia-induced inflammation responses in PD by modulating CX3CL1 release, activation of a single-cell suspension of SN-derived microglia was first confirmed using the CD11b antibody as a broad marker. Flow cytometry showed that the percentage of CD11b-reactive microglia in MPTP-induced PD model mice was significantly increased in the *Park7* KO group compared with that in the WT group, but was significantly reduced by CX3CL1 injections (**Figure 4A** and **B**). To investigate whether neuronal *Park7* reprograms the microglia phenotype through CX3CL1 release, we used flow cytometry to analyze the phenotypic conversion in BV2 microglial cells co-cultured with SH-SY5Y neurons, employing CD86 as the M1 marker and CD206 as the M2 marker (Zhou et al., 2022). As shown in **Figure 4C** and **D**, the M1 marker CD86 was elevated in *Park7* KD cells compared with that in WT cells, but was reduced by the addition of exogenous CX3CL1, inhibiting the shift toward the proinflammatory M1 phenotype. Similarly, *Park7* KD cells exhibited a decrease in the M2 phenotype marker CD206 that was reversed by the addition of exogenous CX3CL1, promoting a shift toward the anti-inflammatory M2 phenotype.

M1-like activation leads to the production of proinflammatory cytokines, including tumor necrosis factor (TNF)-α, IL-1β, and IL-6, which significantly contribute to neurodegeneration in PD (Li et al., 2022; Liu et al., 2022). We used qPCR and ELISAs to analyze the alteration in proinflammatory cytokine expression in tissue lysates of the SN from MPTP-induced PD mice, with and without CX3CL1 intervention. The group treated with CX3CL1 showed markedly reduced mRNA expression levels of TNF-α (*P* < 0.01), IL-6 (*P* < 0.01), and IL-1β (*P* = 0.05) compared with the *Park7* KO group (**Figure 4E–G**). Similarly, ELISAs showed that CX3CL1 significantly reduced the production of IL-1β (*P* < 0.001), IL-6 (*P* < 0.05), and TNF-α (*P* < 0.05) in the MPTP-induced *Park7* KO group compared with the findings in the no CX3CL1 treatment group, correspondingly (**Figure 4H–J**). The production levels of proinflammatory cytokines in MPTP-induced *Park7* KO mice were highly increased comparable to those observed in MPTP-induced WT mice.

### CX3CL1 protects against dopamine cell loss and neurodegeneration

**Figure 5:**
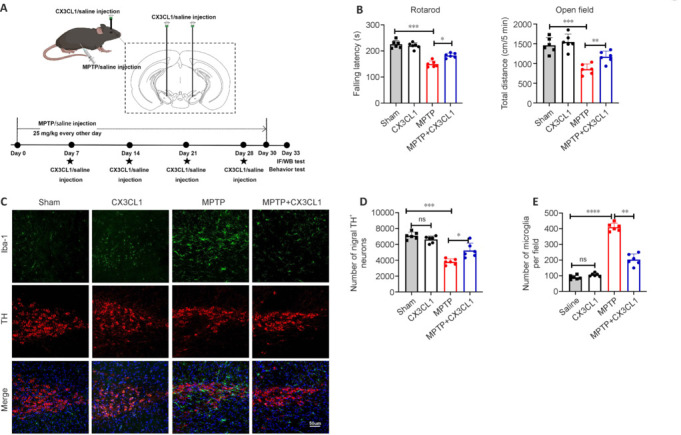
CX3CL1 mitigates MPTP-induced motor deficits, neurodegeneration, and microglial activation. (A) Schematic diagram illustrating the timeline of i.p. injections of MPTP or saline and bilateral injections of CX3CL1 or saline into the SNc for *in vivo* experiments in four treatment groups of mice. (B) Falling latency in accelerating rotarod tests and total distance traveled in open-field tests of motor performance in all four groups. (C–E) Immunostaining and quantification of TH-positive neurons (red, Alexa Fluor 594) and Iba1-reactive microglia (green, Alexa Fluor 488) in the SN of each group. Data expressed as means ± SEM (*n* = 6 per group); *P < 0.05, **P < 0.01, ***P < 0.001, ****P < 0.0001, analyzed by one-way analysis of variance followed by Tukey’s *post hoc* test. CX3CL1: Chemokine (C–X3–C motif) ligand 1; Iba-1: ionized calcium binding adapter molecule; MPTP: 1-methyl-4-phenyl-1,2,3,6-tetrahydropyridine; NC: negative control; ns: no significance; SNc: substantia nigra pars compacta TH: tyrosine hydroxylase.

Next, we designed an experiment to explore the potential protective effects of CX3CL1 on MPTP-induced motor deficits using the accelerating rotarod and open field tests (**Figure 5A**). Four days post-final MPTP injection, the MPTP group showed marked deficits in motor performance, with significant reductions in falling latency and total distance traveled relative to those in the Sham group (*P* < 0.001; **Figure 5B**). CX3CL1 treatment effectively mitigated the MPTP-induced behavioral impairments, with the MPTP + CX3CL1 groups showing significant improvement compared with the untreated MPTP group. To investigate these neuroprotective effects, we quantified TH-positive neurons in the SN as a measure of MPTP-induced neurodegeneration. While the MPTP group showed a substantial reduction in TH-positive neurons compared with the Sham control group, the MPTP+CX3CL1 group exhibited significant preservation of TH-positive neuronal terminals in the SN (*P* < 0.05; **Figure 5C** and **D**). Furthermore, mice that received CX3CL1 injections showed a significant decrease in the number of Iba1-reactive microglia in the SN (*P* < 0.001; **Figure 5C** and **E**).

## Discussion

The findings of this study highlight the crucial role of DJ-1 in shaping microglial polarization and controlling inflammatory responses via CX3CL1 secretion, maintaining neuron–microglia communication in PD models. The absence or knockdown of *Park7* led to a reduction in CX3CL1 levels, which in turn enhanced the release of proinflammatory cytokines. Notably, CX3CL1 treatment was found to shift microglial polarization towards an anti-inflammatory (M2) phenotype while suppressing the proinflammatory (M1) state in *Park7* KO mice subjected to MPTP-induced PD. Through selective knockdown of ADAM10 and ADAM17, we further demonstrated that DJ-1 plays a crucial role in the maturation of ADAM10 within the neuronal ER. This finding identified ADAM10 as the primary protease responsible for CX3CL1 membrane shedding. Collectively, our results suggested that DJ-1 regulates CX3CL1 expression in neurons within PD models and highlighted ADAM10 as the key enzyme mediating the release of CX3CL1 from human neurons.

The reciprocal interactions between microglia and neurons play a fundamental role in the occurrence and development of PD. Microglia, the resident immune cells of the CNS, constantly communicate with neurons to maintain homeostasis and respond to pathological changes (Yang et al., 2025). Multiple studies have presented compelling evidence indicating that microglial functions are influenced by neuronal activities through specific signaling pathways, notably the CD200, CD200 receptor (CD200R), and CX3CL1-CX3CR1 signaling axes. Neurons also promote the extension of microglial processes through a mechanism that involves the activation of neuronal N-methyl-D-aspartate receptors. This activation leads to the release of ATP from neurons, subsequently triggering a microglial response mediated by the P2Y12 member of the P2Y family of G protein-coupled receptors (Dissing-Olesen et al., 2014; Eyo et al., 2014). Although there is substantial evidence for DJ-1 protein regulating the microglial phenotype via inhibition of the NLRP3 inflammasome or cyclic GMP-AMP synthase/stimulator of interferon genes (cGAS/STING) pathways, *Park7* deficiency alone is insufficient to cause PD (Kim et al., 2005). These interactions are critical for modulating microglial activation states and responses to neuronal distress.

DJ-1 is a versatile protein with roles in managing oxidative stress, maintaining mitochondrial function, and modulating inflammatory responses (De Bartolo et al., 2024). It is highly expressed in cells with elevated energy requirements, such as dopaminergic neurons, where it contributes to oxidative stress regulation by stabilizing the Nrf2 protein (Clements et al., 2006). Beyond its antioxidant properties, growing evidence indicates that DJ-1 also has a significant anti-inflammatory role in the brain. Chien et al. (2016) found that the absence of DJ-1 intensified inflammation in the CNS, resulting in heightened microglial activation and reduced viability of dopaminergic neurons, the primary neuronal population affected in PD. DJ-1 was shown to regulate microglial activity by suppressing prolonged STAT1 phosphorylation in response to interferon-gamma, a process mediated through its facilitation of the Src homology 2 domain-containing phosphatase-1 (SHP-1) and STAT1 interaction (Kim et al., 2013). In the absence of *Park7*, microglia showed enhanced mitochondrial activity, leading to a significant rise in the levels of reactive oxygen species compared with control microglia. When stimulated with lipopolysaccharide, *Park7* KO microglia produced increased amounts of proinflammatory cytokines while exhibiting notably reduced expression of the anti-inflammatory protein triggering receptor expressed on myeloid cells 2 (TREM2) (Trudler et al., 2014).

Further studies have shown that the *Park7* KO potentiates the proinflammatory response in microglial cells through the Janus kinase (JAK)-STAT) inflammatory signaling pathway. This enhancement of the inflammatory response extends to astrocytes, another glial cell type, via the p38 mitogen-activated protein kinase (MAPK) signaling pathway (Kim et al., 2013). Astrocytes, which support and protect neurons, also contribute to the inflammatory milieu when dysregulated. *Park7*^–/–^ microglia have been shown to exhibit increased dopaminergic neurotoxicity, manifesting as an abnormal proinflammatory phenotype (Lin et al., 2021). This detrimental phenotype was alleviated by rasagiline, a monoamine oxidase inhibitor approved for the treatment of PD, highlighting potential therapeutic avenues for mitigating inflammation-driven neurodegeneration (Trudler et al., 2014). Additionally, *Park7* deficiency in astrocytes was found to reduce the expression of prostaglandin D2 synthase, an enzyme responsible for the production of prostaglandin D2 (Choi et al., 2019). Prostaglandin D2 is involved in anti-inflammatory processes, and its reduction under *Park7* loss attenuates the anti-inflammatory functions of astrocytes (Choi et al., 2019). Thus, *Park7* deficiency disrupts the balance of pro- and anti-inflammatory signals within the CNS, exacerbating neuroinflammation and contributing to neurodegeneration. Therefore, it is evident that DJ-1 regulates brain inflammation via two mechanisms: direct regulation of proinflammatory mediator expression in microglia and indirect regulation of anti-inflammatory functions in astrocytes. By unraveling these mechanisms, we have gained a deeper understanding of how genetic factors interact with neuroinflammatory processes in PD, opening new avenues for the design of targeted interventions to regulate inflammation and safeguard neuronal integrity.

Hickman et al. (2018) and Jones et al. (2010) demonstrated that exogenous CX3CL1 effectively mitigates neuronal cell death in the striatum while significantly reducing the presence of activated microglial cells. This finding indicates that CX3CL1 has a neuroprotective role and can mitigate the activation of microglia, which are immune cells associated with inflammation and neurodegeneration in the brain. Similarly, Nash et al. (2015) reported positive outcomes from the overexpression of CX3CL1 in a PD model featuring the A53T α-synuclein mutation. Their findings underscored the therapeutic potential of enhancing CX3CL1 expression to counteract neurodegeneration associated with PD. Furthermore, Morganti et al. (2012) conducted a detailed study showing that only the soluble isoform of CX3CL1 was effective in reducing the neurotoxic effects of MPTP, which is commonly used to model PD. This study underscores the importance of the soluble form of CX3CL1 in providing neuroprotection. The beneficial effects of CX3CL1 were further supported by Tristão et al. (2016), who demonstrated that CX3CL1 led to improved motor coordination, a reduction in lesion size, decreased activation of microglia, lowered levels of proinflammatory cytokines, and protection of dopaminergic neurons in the SN. These findings collectively emphasize the importance of CX3CL1 in maintaining neuronal health and reducing brain inflammation. However, in the study conducted by Nash et al. (2015), the membrane-bound form of CX3CL1 failed to demonstrate neuroprotective effects, highlighting a functional disparity between the soluble and membrane-bound isoforms. Conversely, Lyons et al. (2009) suggested that the membrane-bound variant exhibits anti-inflammatory properties akin to those of the soluble forms. This claim, however, remains a topic of debate because their research did not evaluate the effects of inhibiting the cleavage mechanism by which the membrane-bound form is converted into its soluble counterpart, leaving room for further investigation. Extensive evidence indicates that this proteolytic cleavage of CX3CL1 is a crucial regulatory mechanism governing its activity *in vivo*. This process is likely essential for modulating the functional roles of CX3CL1 in physiological and pathological contexts. Understanding and targeting this regulatory mechanism may be key to harnessing its therapeutic potential in neurodegenerative diseases.

Variations in CX3CL1 signaling pathways are likely influenced by the unique attributes of the experimental models employed and the specific triggers of the degenerative processes under investigation. Castro-Sánchez et al. (2018) reported that the degeneration of dopaminergic neurons was exacerbated, and the production of proinflammatory markers was increased, in CX3CL1 KO (*CX3CL1*^–/–^) mice. This finding suggests that the absence of CX3CL1 contributes to heightened inflammation and worsened neuronal degeneration. Interestingly, Lastres-Becker et al. (2014) found that a lack of the CX3CL1/CX3CR1 signaling axis did not result in the loss of nigral dopaminergic neurons in mice treated intranasally with MPTP or 6-hydroxydopamine. This indicates that, under certain conditions, the absence of CX3CL1 signaling might not lead to neurodegeneration, suggesting a context-dependent role of this pathway. In contrast, Tristão et al. (2016) reported opposing findings, demonstrating that the absence of the CX3CL1/CX3CR1 axis exacerbated the damage caused by i.p. administered MPTP. Such conflicting results highlight the complexity of CX3CL1 signaling, whose effects on neuroinflammation and neurodegeneration appear to depend on the mode of toxin delivery and possibly other experimental conditions. Collectively, these findings suggest that, while CX3CL1 signaling is a key regulator of neuroinflammatory processes in PD models, its effects are multifaceted and heavily influenced by the specific conditions and triggers of neurodegeneration. Further research is needed to investigate the potential protective role of CX3CL1 in preventing neuroinflammation associated with PD. Understanding the nuances of CX3CL1 signaling could lead to novel therapeutic interventions that target this pathway to mitigate inflammation and protect neuronal health in neurodegenerative diseases.

The involvement of ADAM10 and ADAM17 in the development of neurodegenerative diseases, including Alzheimer’s disease, has been highlighted by Allinson et al. (2003) and Manzine et al. (2013). ADAM10 is widely expressed throughout the CNS, whereas ADAM17 expression is more restricted, according to Kärkkäinen et al. (2000). The extensive presence of ADAM10 throughout the CNS indicates its likely involvement in the continuous, baseline processing of ligands such as CX3CL1. By contrast, the more limited expression of ADAM17 aligns with its suggested role in regulated, stimulus-driven proteolytic activity, as observed by Lammich et al. (1999). Hundhausen et al. (2003) first identified ADAM10 as a key enzyme involved in the constitutive cleavage of CX3CL1 in mouse embryonic fibroblast cell lines. Similarly, O’Sullivan et al. (2016) attributed the cytokine-induced proteolytic processing of soluble CX3CL1 to the activity of ADAM10. Here, we have identified ADAM10 as the primary enzyme facilitating CX3CL1 shedding in neurons. This conclusion is supported by Hurst et al. (2012), who showed that reducing ADAM10 expression via siRNA significantly decreased CX3CL1 levels in a human adult brain endothelial cell line exposed to proinflammatory cytokines, whereas knockdown of ADAM17 had no such effect. This finding implies that the regulation of ADAM10 and ADAM17 varies depending on the cell type, potentially enabling distinct cellular responses to identical stimuli. Specifically, while ADAM10 may be primarily responsible for the routine, baseline processing of certain proteins, ADAM17 might be activated under specific conditions to mediate inducible processing. This cell type-specific regulation could be crucial for understanding how different cells within the CNS respond to inflammatory stimuli and contribute to the pathogenesis of neurodegenerative diseases.

In summary, our findings elucidated that DJ-1 plays a crucial role in modulating microglial polarization to counteract the inflammatory response in PD models. The CX3CL1 pathway is central to maintaining microglial activation. To better understand this process, future studies should focus on how the DJ-1/CX3CL1/CX3CR1 axis influences the microglial phenotypic changes driven by dopaminergic neurons. Shi et al. (2011) previously reported that the ratio of CX3CL1 to Aβ42 in cerebrospinal fluid (CSF), along with CX3CL1 levels in the CSF, showed a strong correlation with disease progression, particularly in rapidly progressing PD patients observed in a longitudinal study. Our research corroborates these findings, showing increased levels of CX3CL1 in brain samples from PD patients and dynamic changes in serum CX3CL1 levels that correspond to different stages of disease severity. Contrary to these findings, Gui et al. (2015) identified significantly lower levels of CX3CL1 mRNA in exosomes isolated from the CSF of PD patients compared with the levels in those from the CSF of HCs. Interestingly, during disease progression, CSF CX3CL1 levels appeared to rise. This increase may reflect one of two things: enhanced activation of microglia due to dopaminergic neuron loss, or a protective adaptation by the remaining neurons aimed at preventing further damage and regulating microglial overactivation. To address these conflicting findings, comprehensive studies involving simultaneous measurement of CX3CL1 concentrations in the CSF at both the mRNA and protein levels are needed, using larger cohorts of PD patients and HCs. This approach will help clarify the complex relationship between CX3CL1 levels and disease progression, providing a deeper understanding of the mechanisms behind PD and potentially guiding the development of new therapeutic strategies.

The limitations of this study include its reliance on PD models, which may not fully replicate the complexity of human PD pathology. Additionally, the specific experimental conditions employed here may not have encompassed all of the regulatory mechanisms of the DJ-1/CX3CL1/CX3CR1 axis used *in vivo*. Furthermore, the study did not address how external factors, such as genetic or environmental modifiers, may influence the DJ-1/CX3CL1 pathway. Lastly, longitudinal studies with larger patient cohorts are necessary to confirm the clinical relevance of CX3CL1 as a therapeutic target in PD.

Our study proposes a novel role for DJ-1 in mediating CX3CL1/CX3CR1 signaling to influence microglial activation and neuroinflammatory responses in PD models. Previous research has implicated ADAM10 in the cleavage of CX3CL1 from neurons. Further *in vivo* studies in PD models are crucial to unravel the complex mechanisms underlying neuron–microglia interactions and their role in driving neuroinflammation. Our findings highlight CX3CL1 as a potential therapeutic target for addressing neuroinflammatory pathways in PD, emphasizing its clinical significance. Investigating how CX3CL1 influences microglial behavior may open new avenues for developing treatments aimed at slowing disease progression and improving patient outcomes in PD.

## Data Availability

*No additional data are available*.
